# Can Transcriptomics Cut the Gordian Knot of Amyotrophic Lateral Sclerosis?

**DOI:** 10.2174/138920211797904043

**Published:** 2011-11

**Authors:** Alexandre Henriques, Jose-Luis Gonzalez De Aguilar

**Affiliations:** 1 INSERM, U692, Laboratoire de Signalisations Moléculaires et Neurodégénérescence, Strasbourg, France; 2 Université de Strasbourg, UMRS692, Strasbourg, France

**Keywords:** Amyotrophic lateral sclerosis, biomarker, pathogenesis, transcriptomics.

## Abstract

Amyotrophic lateral sclerosis (ALS) is an adult-onset degenerative disease characterized by the loss of upper and lower motor neurons, progressive muscle atrophy, paralysis and death, which occurs within 2-5 years of diagnosis. Most cases appear sporadically but some are familial, usually inherited in an autosomal dominant pattern. It is postulated that the disease results from the combination of multiple pathogenic mechanisms, which affect not only motor neurons but also non-neuronal neighboring cells. Together with the understanding of this intriguing cell biology, important challenges in the field concern the design of effective curative treatments and the discovery of molecular biomarkers for early diagnosis and accurate monitoring of disease progression. During the last decade, transcriptomics has represented a promising approach to address these questions. In this review, we revisit the major findings of the numerous studies that analyzed global gene expression in tissues and cells from biopsy or post-mortem specimens of ALS patients and related animal models. These studies corroborated the implication of previously described disease pathways, and investigated the role of new genes in the pathological process. In addition, they also identified gene expression changes that could be used as candidate biomarkers for the diagnosis and follow-up of ALS. The limitations of these transcriptomics approaches will be also discussed.

## A BRIEF OVERVIEW ON ALS

Amyotrophic lateral sclerosis (ALS), also referred to as motor neuron disease or Lou Gehrig’s disease, is a fatal condition caused by the selective degeneration of upper motor neurons, which originate in the motor cortex and give rise to the pyramidal tract, and lower motor neurons, which connect the spinal cord and brainstem to skeletal musculature. Typical clinical features include progressive muscle weakness and atrophy, fasciculations, hyperreflexia, dysarthria and dysphagia. ALS most commonly develops between 40 and 70 years of age, and affects about two per 100,000 people. Death often occurs by respiratory complications within two to five years of diagnosis [1]. About 90% of cases are sporadic, since they are not associated with a documented family history. The remainder cases are inherited, usually in an autosomal dominant pattern. Sporadic and familial forms of the disease are clinically and pathologically undistinguishable, suggesting common pathogenic mechanisms [2]. Riluzole, which is postulated to fight against motor neuron death by preventing glutamate-induced excitotoxicity, is the only approved medication for the treatment of ALS but merely prolongs patient’s survival for several months [3].

Numerous genetic factors have been shown to predispose to ALS or to be associated with (mostly atypical) forms of the disease [http://alsod.iop.kcl.ac.uk/]. In addition, specific mutations have been found in a small group of genes that cause directly ALS. These genes encode a variety of proteins, such as the antioxidant enzyme Cu/Zn superoxide dismutase (SOD1) [4], and the RNA processing regulatory factors TAR DNA binding protein 43 (TDP-43) [5] and fused in sarcoma/translocated in liposarcoma (FUS/TLS) [6]. The etiology of the selective degeneration of motor neurons in ALS, whatever familial or sporadic, still remains elusive. Many pathogenic mechanisms have been shown to be involved, including protein misfolding [7], mitochondrial dysfunction [8], oxidative damage [9], altered DNA/RNA processing [10], energy imbalance [11], defective axonal transport [12], excitotoxicity [13], insufficient growth factor signaling [14] and inflammation [15]. During the last years, the situation has become more complex than imagined, because it is postulated that the pathogenic process implicates not only motor neurons but also non-neuronal neighboring cells, such as astrocytes [16] and microglial cells [17] and, beyond the central nervous system, skeletal myocytes [18] and, likely, other cells in the body.

The diagnosis of ALS is exclusively based on clinical examination, supportive electrophysiological findings, medical history, and exclusion of confounding disorders that mimic the disease [19]. During the early stages of ALS, not all patients present with the same symptoms, which usually start at one point and then spread to other parts in the body in a quite variable manner from one person to another. Only the worsening of the symptoms and signs indicating both upper and lower motor neuron pathology can assess the presence of ALS. In average, the confirmation of diagnosis from the onset of symptoms takes 13–18 months [20]. There is therefore an unmet need for finding specific and sensitive molecular markers (or biomarkers), which could help with earlier diagnosis and more precise monitoring of disease progression.

During the last few years, numerous “omics” approaches have attempted to identify from a global perspective changes in the expression of genes, or in the amount of proteins and metabolites that could shed light into the pathogenesis of ALS or be used as biomarkers of disease [21-23]. Particularly, studying the transcriptome allows to determine the combined expression of thousands of genes in organs, tissues or cells at different stages of a given pathology. At the time of first transcriptomics studies on ALS, the technology was at its beginning and only capable of screening between hundreds and several thousands of transcripts. The sophistication of the microarrays rapidly enhanced their ability to measure at once more than 30,000 transcripts. Nowadays, one can obtain a complete overview of the expression of the entire genome, by analysing not only whole genes but also their related splice variants. These technological improvements have resulted in detecting more and more significant expression changes, affecting less than a hundred of genes in pioneering studies [23-25] and more than 2,000 in the latest ones [26, 27]. The exponential increase in the number of genes that appear differentially expressed in ALS points out the complexity of the disease and the diversity of the pathways involved in promoting or preventing neurodegeneration. These findings have been accompanied by a shift in the conceptual understanding of ALS. While initial studies were restricted to the analysis of gene expression changes in whole tissue samples from the central nervous system (CNS) [28, 29], follow-up studies refined their experimental designs to better decipher the nature of the puzzling non-cell autonomous paradigm of ALS [30, 31]. The objective of this review is to revisit the numerous studies that analysed the gene expression profiles of tissues and cells from biopsy or post-mortem specimens of ALS patients and related animal models. Such studies provided insight into previously described disease mechanisms, and investigated the role of new genes involved in the pathological process. In addition, promising results were obtained for several potential biomarkers, which could be used in clinics. The limitations of these transcriptomics approaches will be also discussed.

## TRANSCRIPTOMICS FOR THE ANALYSIS OF ALS PATHOGENESIS

### Gene Profiling in Post-Mortem Human ALS Tissue

**Fig. (1) Major gene expression changes in ALS.**
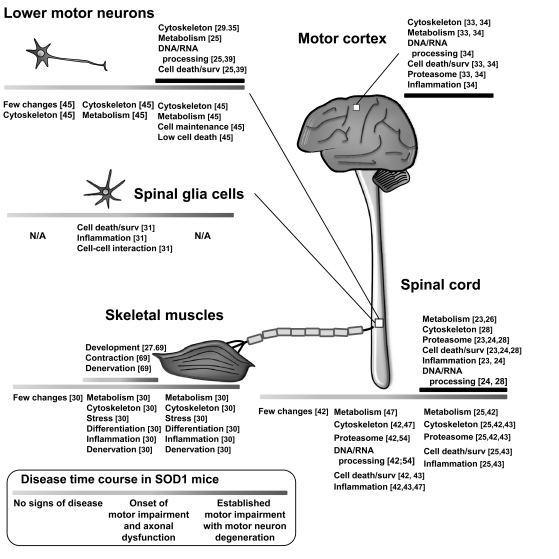
Schematic representation of the most important findings from studies performed on human ALS and their related mutant SOD1 transgenic models. Upper lines represent disease progression in patients, and lower lines represent disease progression in animals. Gene functional categories are indicated for different tissues or cells. The inset shows the progression of the cellular events and clinical symptoms corresponding to each pathological stage in the mouse (pre-symptomatic, early symptomatic and late symptomatic). N/A, not available; surv., survival; comm., communication.

The first ALS transcriptomics study was released in 2001 and concerned patients presenting with the sporadic form of the disease [23]. This study, as well as others undertaken since then, focused on the understanding of the process of motor neuron death as such, using autopsy specimens of mainly spinal cord [23, 24, 28, 29, 32] but also motor cortex [33, 34]. The generated gene expression profiles showed the activation of several mechanisms classically associated with ALS at disease endpoint [2], including activation of cell death and survival pathways [23, 28, 29, 33-35], response to oxidative stress [23, 24, 28, 33, 34] and inflammation [23, 24, 33, 34], and protease activity [23, 24, 28, 33, 34] (Fig. **1**).

In addition, these studies also showed the implication of new pathways relevant to ALS and, in some cases, predicted later observations obtained by other means. For instance, expression changes were found in the CNS for genes involved in lipid metabolism [23, 34-36], including 25-cholesterol hydroxylase [23], apolipoprotein E [36] and oxysterol binding protein-like 11 [34] genes, which suggests that cholesterol metabolism is altered in ALS. Interestingly, strong evidence now supports the role of a defective energy metabolism at the whole body level in the onset and progression of ALS [18] and, in particular, it has been shown that hyperlipidemia is a significant prognostic factor for patient’s survival [37]. Transcriptomics studies on human CNS also highlighted changes in the expression of genes related to axonal transport and the cytoskeleton [28, 29, 33-35]. Among these, the expression of dynactin and dynein, which play a key role in intracellular trafficking, was found to be down-regulated [29, 35]. Dynactin dysfunction might be detrimental to motor neurons as it would block axonal transport [38]. Curiously, a mutant dynactin gene variant has been found to occur in familial cases of ALS [39]. Another important conclusion of the CNS gene expression profiles was the alteration of the processing of RNA [23, 24, 28, 29, 35], which is in agreement with recent discoveries reporting the identification of mutant forms of TDP-43 and FUS/TLS, both being involved in RNA processing, in several pedigrees of ALS patients [40]. 

### Gene Profiling in SOD1(G93A) Transgenic Rodent Models of ALS

The findings based on post-mortem human ALS tissues cannot ascertain whether altered gene expression is a secondary marker of neurodegeneration or the cause of it. In contrast, animal models of disease allow to collect samples not only at every step of the pathological process but also during the pre-symptomatic phase, when initiatory pathogenic events can arise at the molecular level. Moreover, the quality of RNAs extracted from animal samples is generally higher as delay between sacrifice and collection is normally short and can be easily controlled. A subset of patients with autosomal dominantly inherited ALS harbor point mutations in the gene encoding SOD1, which is a free radical scavenging enzyme that protects cells against oxidative stress [4]. The identification of these mutations allowed the production of transgenic mice overexpressing different mutated SOD1 genes. Nowadays, the SOD1(G93A) strain is the most commonly used model of ALS. These mice develop a precipitous, age-related loss of motor neurons, together with related clinical symptoms (i.e. altered hind limb extension reflexes, muscle weakness and paralysis), that strongly resembles human ALS, and represent a valuable model to investigate the molecular mechanisms underlying the disease [41].

In the spinal cord of pre-symptomatic SOD1(G93A) mice, gene expression changes appeared modest [25, 42, 43], and were mostly concerned with metabolic alterations [44], mitochondrial dysfunction [44], and axon and cytoskeleton disturbances [42]. Of note, the expression of the intermediate filament vimentin was consistently found to be up-regulated in several studies [25, 42, 45]. Vimentin plays an important role in the organization of the cytoskeleton and interacts with dynein to mediate neurite outgrowth [46]. Using laser-captured microdissected spinal motor neurons, Perrin *et al*. [45] showed that the up-regulation of vimentin mostly occurred within these cells, and postulated that its aggregation might lead to neurodegeneration. In the spinal cord of SOD1(G93A) mice at an early stage of disease, when first symptoms arrive, the gene profiles revealed that the changes already detected during the pre-symptomatic phase gained in intensity [25, 42-44]. In addition, other cellular processes appeared affected at the transcriptional level, including mainly the response to oxidative stress and inflammation and, to a lesser extent, pro-survival mechanisms, proteasome activity, and RNA processing. Curiously, the expression of genes involved in cell death pathways was not significantly up-regulated specifically within motor neurons, indicating that the loss of these cells is a late event in the disease [44, 45]. Finally, the pattern of gene expression in the spinal cord of SOD1(G93A) mice at end stage was very similar to that found in post-mortem specimens of human ALS, and affected genes involved in cell death [25, 43, 47] and neuroprotection [43, 44], inflammation [25, 43, 47], proteasome function [25, 42, 43], metabolism [25, 42, 47], cytoskeleton and axonal guidance [25, 42-44, 47] (Fig. **1**).

It is now commonly accepted that motor neuron degeneration in ALS is the result of the combined participation of several cell types, including the motor neurons themselves and other non-neuronal neighboring cells [2]. Thus, it can be envisaged that the transcriptional profile of motor neurons, whatever embedded into the spinal cord or isolated by microdissection, is imprinted by their cellular environment. Consequently, it cannot be clearly established whether particular gene expression changes are intrinsically motor neuron-specific or the consequence of cell-to-cell interactions. Several studies prompted to bypass this difficulty by analysing the transcriptome of cells, including cortical neurons [48] and spinal cord astrocytes [49], which were isolated from SOD1(G93A) mouse and rat embryos, respectively, and then kept in culture. Other studies preferred to use NSC34 [50] or SH-SY5Y [51] neuronal cell lines transfected with mutant SOD1(G93A). In general, many of the expression changes detected by these approaches were similar to those found in the human and animal studies.

Together with the studies that focused on the progression of disease in SOD1(G93A) mice, it is noteworthy that the use of this animal model also allowed to analyze the transcriptome in response to induced stress, which can give access to potential intrinsic deficiencies owing to mutant SOD1 toxicity. Recently, Jokic *et al*. [52] investigated the gene expression profile of the spinal cord in SOD1(G93A) rats in response to mild compression. Shortly after injury, the expression of genes involved in inflammation and apoptosis was significantly increased while that of genes related to angiogenesis appeared reduced. Later on 7 days post-injury, the expression pattern was characterized by down-regulation of pro-survival genes and up-regulation of genes implicated in axonal transport and metabolic pathways. It was proposed that mutant SOD1 predisposes to functional impairment that becomes visible, already at a pre-symptomatic age, under particular stress conditions.

### Gene Profiling in other Mutant SOD1 Transgenic Models of ALS

A new transgenic line, the SOD1 L126 delTT mouse, was generated by expressing a truncated form of SOD1 that leads to pathological features and motor neuron degeneration similar to that observed in ALS [53]. Recently, Fukada *et al*. [54] analyzed the transcriptome in whole spinal cord extracts from pre-symptomatic and diseased animals, and found that the most important changes in gene expression were related to inflammation. Moreover, changes were modest in pre-symptomatic mice, and concerned genes involved in RNA processing and protein synthesis. At disease stage, an increased number of expression changes affected genes implicated in inflammation and neuroprotection but also protein degradation and cell death. Interestingly, immunohistochemistry validation of several of these gene expression changes revealed that such transcriptional regulations came from glial cells and not motor neurons, which is consistent with the marked inflammatory trait observed in this mouse line.

Although still debated, growing evidence suggests that primary muscle abnormalities may occur in ALS. Muscle restricted expression of mutant SOD1 was sufficient to trigger muscle atrophy and mitochondrial dysfunction [55]. In addition, mild uncoupling of mitochondrial respiration in skeletal muscle was sufficient to affect neuromuscular junction stability and function, and induce distal degeneration of motor neurons [56]. A recent study used SOD1(G86R) mice, which harbour another point mutation in the SOD1 gene different from that of the G93A line [57], and characterised the gene profile in skeletal muscle during the course of ALS [30]. As previously exposed in the case of the spinal cord, only modest gene expression changes were found in the muscle of pre-symptomatic SOD1(G86R) mice. In contrast, significant transcriptional changes appeared at the moment of first clinical symptoms (i.e. altered hind limb extension reflexes), affecting genes mostly involved in cell cycle, growth and differentiation, cytoskeleton, and the response to stress and inflammation. At the onset of hind limb paralysis, the whole transcriptome changes gained in intensity, indicating advanced atrophy. Because motor neuron loss is a late event in SOD1(G86R) mice, the progression of the gene expression changes detected in muscle was consistent with the notion that the degeneration of lower motor neurons proceeds in a distal to proximal dying-back pattern.

### Gene Profiling in Transgenic Mouse Models of ALS Unrelated to Mutant SOD1

Initially generated to investigate the effects of hypoxia on the biological functions of vascular endothelial growth factor (VEGF), VEGF-delta/delta mice harbor a deletion of the hypoxia-responsive element in the promoter sequence of the VEGF gene. Surprisingly, the phenotype of these mice closely resembles that of ALS, including the presence of muscle weakness, axonal degeneration and spinal motor neuron loss. However, such an ALS-like syndrome is not progressive, and the lifespan of VEGF-delta/delta mice is not compromised. Using laser-captured microdissected spinal motor neurons from this animal model, Brockington *et al*. [58] showed that the gene profile in VEGF-delta/delta mice at the symptomatic stage was quite similar to that observed in diseased SOD1(G93A) mice. In contrast, important expression changes concerning genes involved in oxidative metabolism were already detected before disease. It was proposed that some kind of metabolic dysfunction is an early event in this model, which may predispose to perturbed axonal maintenance and subsequent dying-back of motor neurons.

Very recently, two mouse lines overexpressing mutant ALS-linked and wild-type TDP-43 were generated, and the transcriptome in the cortex of these mice was analyzed [26]. The observed expression changes mainly concerned genes involved in response to stimulus and chromatin assembly, which is consistent with the role of TDP-43 in regulating gene expression and splicing events. Interestingly, these transcriptional changes occurred in both mutant and wild-type mice, suggesting that the toxicity of TDP-43 might be due to its accumulation rather than to the fact that the protein was mutated.

### Gene Profiling in Healthy Wild-Type Mice

To better understand the complex pathogenic mechanisms underlying motor neuron death in ALS, several studies preferred to investigate the transcriptome of healthy motor neurons in response to induced stress, or the intrinsic characteristics of different populations of motor neurons as a means to elucidate vulnerability factors. Then, the findings could be extrapolated to the disease condition. The cause-and-effect relationship between physical activity and the development of ALS is a matter of controversy, since it is not well established yet whether some kind of exercise could be considered either as a risk factor for the disease [59] or beneficial, at least in the animal [60]. Ferraiuolo *et al*. [61] studied the gene profiles of isolated spinal motor neurons and skeletal muscle after submitting the mice to physical activity in the form of running-like exercise. Major expression changes in motor neurons concerned genes implicated in growth factor pathways and axonal transport. Genes involved in neovascularization and myogenesis appeared regulated in the gastrocnemius muscle. It was suggested that deficiencies in these cellular processes in one or both compartments of the motor unit might lead to a perturbed response to mechanical stress and subsequent neuromuscular pathology. Another study also addressed this question by analyzing the transcriptome of the whole spinal cord of mice subjected to physical activity [62]. In this case, genes involved in metabolism, and transcription regulation were up-regulated while genes involved in glutamate signaling were down-regulated. Statistical testing was however not performed, so that proper conclusions could not be obtained.

In ALS, motor neurons in the spinal cord and motor cortex are more prone to degeneration than motor neurons in the oculomotor nucleus, the function of which remains quite preserved during the whole disease process [63]. To better understand the reasons of such a discrepancy, Hedlund *et al*. [64] undertook the characterization of the transcriptome of these two subtypes of motor neurons in healthy animals. Major differences in gene expression between spinal and oculomotor motor neurons concerned pathways known to be involved in the physiopathology of ALS, such as mitochondrial dysfunction, altered RNA processing and perturbed axonal transport. Of note, it was found that the expression of peripherin, the aggregation of which has been related to axonal dysfunction in ALS [65], was higher in spinal motor neurons than oculomotor motor neurons. The latter, in contrast, showed an enhanced expression of genes with protective functions, such as growth factors, suggesting a higher stimulation of autocrine neuroprotective pathways. These findings indicated that spinal motor neurons might be intrinsically more sensitive to stress and subsequent disease.

## SEARCHING FOR BIOMARKERS OF ALS BY TRANSCRIPTOMICS

The use of transcriptomics as a means to determine gene expression changes of diagnostic or prognostic value represents a promising approach for the management of ALS patients, since the identification of such a kind of biomarkers would greatly help to get earlier diagnosis and to monitor more precisely disease progression, not only in clinical practice but also in the context of therapeutic trials. Several studies have addressed these questions by analyzing the transcriptome of peripheral blood cells and skeletal muscles (Table **1**), which allow easy or relatively mild invasive access for sampling.

Using peripheral blood cells from patients with sporadic ALS, Saris *et al*. [66] found important changes in the expression of genes mainly involved in RNA processing and mitochondrial function. They generated two subsets of candidate biomarkers, each containing several hundreds of genes, that showed up- or down-regulated expression, respectively, as compared to that observed in a group of healthy subjects. Interestingly, no significant relationship was found between the detected gene expression changes and several clinical variables, including age, gender and site of onset of disease (that is, bulbar versus spinal). These findings prompted the authors to propose that such a persistent differential gene expression could be specific to ALS. Another study that focused on the activation of peripheral monocytes/macrophages in ALS [67] revealed the increased expression of a series of genes implicated in the pro-inflammatory LPS/TLR4 signaling pathway. These gene expression changes occurred independently of disease duration and clinical status, except for the expression of alpha-1-acid glycoprotein, the increase of which correlated with the ALSFRS-R score, a functional rating scale that assesses the activities of daily living in patients with ALS. 

An original approach was presented by Kudo *et al*. [31], who compared the pre-symptomatic transcriptomes of motor neurons and glial cells between SOD1(G93A) mice and Tau P301L mice, which is a model of neurodegeneration showing typical ALS-like features (including loss of motor neurons, reactive gliosis, and muscle weakness and atrophy), as well as other degenerative signs in the cerebellum, hypothalamus and hippocampus [68]. It was assumed that the genes with common differential expression in the two models could be considered as candidate biomarkers of ALS. By this approach, the authors identified three genes, encoding neurofilament heavy chain, peripherin and monoglyceride lipase, as being dysregulated in both CNS and peripheral blood cells of pre-symptomatic animals.

Using muscle biopsies, a recent study attempted to identify disease biomarkers in patients with ALS and multifocal motor neuropathy (MMN), which is a treatable condition that can be mistaken for ALS [27]. The expression of several genes, involved in cell cycle, differentiation, survival and growth, was shown to be up-regulated only in the ALS condition, as compared to a group of healthy subjects. These changes were proposed as candidate biomarkers for diagnosis, but the findings could not be conveniently validated. Another study focused on the identification of gene expression changes that could be markers of disease progression in muscle biopsies from patients with sporadic ALS at different pathological stages [69]. Major changes in differentially expressed genes, as compared to that observed in healthy subjects, concerned neuromuscular transmission and the contractile machinery, as well as muscle development and growth. A set of one hundred and a half genes distinguished patients at an advanced stage from patients with no clinical or electrophysiological signs in the biopsied muscle. Among these, the expression of a reduced subset of nine genes, known also to be regulated in mutant SOD1 mice, discriminated between early and advanced stages of the muscle pathology, as determined by the manual muscle testing, electromyography and histological examination of the degree of muscle atrophy.

## TRANSCRIPTOMICS FOR ALS, RIGHT OR WRONG?

Most of the studies we presented herein placed the microarrays at the center of the work. This approach is evolving, since current studies rather use the transcriptomics technology as part of complex analyses of the pathogenic role of a particular gene, by applying many other experimental means. For example, Cox *et al*. [70] investigated the implication of frontotemporal lobar dementia-linked CHMP2B mutations in lower motor neuron predominant ALS, by combining genetic sequencing, gene profiling and subsequent characterization of the cellular pathways involved. In another example, Fiala *et al*. [71] used the microarrays to confirm the role of previously identified inflammatory factors in ALS induced by mutant SOD1.

Regardless of its relative importance in a given study, transcriptomics still faces a number of problems, which can be summarized as follows:

### (1) Thresholds

Due to the use of necessary but arbitrary thresholds for filtering purposes, many gene expression changes showing low regulations are systematically neglected. In addition, many of these small changes in gene expression will appear as false negative results when the number of samples is too small to allow robust statistical testing. These handicaps can account for the small number of gene expression changes occurring during the pre-symptomatic stages of ALS [42, 43]. Increasing the number of samples might bypass this difficulty.

### (2) Translation

The changes in mRNA levels do not necessarily translate into modifications in protein amounts. Classically, findings from gene profiling studies have been validated by additional sets of experimental approaches, including immunohisto-chemistry [29, 31-33, 35, 45, 52, 54, 71] and Western blot [25, 27, 31, 33, 52]. However, combining transcriptomics with proteomics and/or metabolomics should enrich the interpretation of the results.

### (3) Animal models

Most of transcriptomics data come from the mutant SOD1 mouse model of ALS. Other new models of ALS-like motor neuron degeneration are now available to the scientific community, such as mutant FUS overexpressing rats [72] or p150Glued G59S dynactin mice [73]. Studies on the transcriptome in these animals and comparison with previous data would allow identification of common gene expression changes in a disease-specific manner.

### (4) Study design

Studies that focused on the discovery of biomarkers specific to ALS typically used healthy subjects as controls. From a clinical point of view, however, comparing patients with ALS to patients presenting with confounding disorders should provide more appropriate biomarkers of specificity. In addition, the analysis of the transcriptome in asymptomatic individuals from an ALS pedigree harboring a known causative mutated gene, although ethically challenging, would enable the detection of very early gene expression changes directly linked to the pathogenic mechanism(s) of the disease rather than being the consequence of the pathological process.

### (5) Use of blood cells

One could ask whether blood cells are a valuable source of biomarkers, when considering the neuromuscular nature of ALS. It seems, however, that these peripheral cells take advantage of the expression of essential genes that could be generally affected during disease [66]. Of note, changes in the expression of specific genes have been shown to occur at the peripheral level in the context of other neurodegenerative diseases [66]. In addition, inflammatory events have been reported to take place in ALS [74], a part of which can be found at the periphery [67, 71].

### (6) Use of muscles

Major advantages of using skeletal muscle tissue to search for ALS biomarkers are that this tissue is easily accessible, and is directly affected by the disease, at an early stage that precedes motor neuron death [75]. However, collecting muscle biopsies remains relatively invasive, and is not part of the work-up for the diagnosis of ALS. Moreover, multiple testing on the same patient is often not ethically feasible. In contrast, a needle biopsy technique would offer the opportunity to obtain muscle specimens by a less invasive procedure.

Despite a certain number of limitations, it is concluded that transcriptomics remains a powerful tool to investigate whole gene expression in ALS. Interestingly, the generated databases, even if a priori misunderstood, can provide very valuable information many years later. A good example comes from the first ever transcriptomic microarray in the field. In 2001, the expression of granulin [23] was found to be altered in ALS. Later, it was observed that the granulin gene was linked to frontotemporal lobar dementia [76], which has been shown to share intriguing physiopathological relationships with ALS [40].

## Figures and Tables

**Table 1:** Potential Candidates for Specific Biomarkers for ALS

Study	Tissues	Purpose	Patients	Biomarkers

Saris *et al*. [66]	Blood cells	Diagnostic	123 ALS	Module 1 : 500 upregulated genes
			123 controls	Module 2: 500 downregulated genes

				Interferon alpha-inducible protein 6
				G protein-coupled receptor 43
				Interferon-induced protein with tetratricopeptide repeats 2
Zhang *et al*. [67]	Blood cells	Diagnostic	20 ALS	Alpha-1-acid glycoprotein
			22 controls	Peptidase inhibitor 3
				Chitinase 3-like 1
				Interleukin 1 receptor antagonist
				TNF-related apoptosis inducing ligand

				Sestrin 3
				Collagen, type XIX, alpha-1
			3 ALS	CXorf64
Shtilbans *et al*. [27]	Prognator, gracilis, and gastrocnemius muscles	Diagnostic	3 MMN	Leucine-rich repeat kinase 2
			3 controls	Leucine-rich repeat kinase 2
				Ceramide kinase-like
				Follistatin

				Cholinergic receptor, nicotinic, alpha 1
				Myosin binding protein H
				Myogenin
				Ras-related associated with diabetes
			9 ALS	Cholinergic receptor, nicotinic, gamma
Pradat *et al*. [69]	Deltoid muscle	Prognostic	10 controls	Growth arrest and DNA-damage-inducible protein GADD45 alpha
				Ankyrin repeat domain 1
				Zinc finger protein 36, C3H type-like 2
				Ankyrin repeat domain 10
